# Glutathione S-Transferase M1 (GSTM1) and T1 (GSTT1) Null Polymorphisms and the Risk of Hypertension: A Meta-Analysis

**DOI:** 10.1371/journal.pone.0118897

**Published:** 2015-03-05

**Authors:** Beihai Ge, Yadong Song, Yi Zhang, Xiaowen Liu, Yuxiang Wen, Xiaomei Guo

**Affiliations:** 1 Department of Cardiology, Tongji Hospital, Tongji Medical College, Huazhong University of Science and Technology, Wuhan 430030, China; 2 Department of Nutrition and Food Hygiene, School of Public Health, Tongji Medical College, Huazhong University of Science and Technology, Wuhan 430030, China; University of Insubria, ITALY

## Abstract

**Background:**

Some studies have recently focused on the association between glutathione S-transferase M1 (GSTM1) and glutathione S-transferase T1 (GSTT1) null polymorphisms and hypertension; however, results have been inconsistent.

**Objective:**

In order to drive a more precise estimation, the present systematic review and meta-analysis is performed to investigate the relationship between the GSTM1 and GSTT1 null polymorphisms and hypertension.

**Methods:**

Eligible articles were identified by a search of several bibliographic databases for the period up to August 17, 2013. Odds ratios were pooled using either fixed-effects or random-effects models.

**Results:**

Regarding the GSTM1 null/present genotype, 14 case—control studies were eligible (2773 hypertension cases and 3189 controls). The meta-analysis revealed that it might present a small increased risk for hypertension, although the effect was not statistically significant (odd ratio (OR) = 1.16, 95% confidence interval (CI): 0.96, 1.40; P = 0.002, I2 = 59.8%). Further subgroup analysis by ethnicity and control source suggested that the association was still not significant. Thirteen case—control studies were eligible for GSTT1 (2497 hypertension cases and 3078 controls). No statistically significant association was observed between the GSTT1 null genotype and hypertension risk (OR = 1.14, 95% CI: 0.85, 1.53; P = 0.000, I2 = 80.3%). Furthermore, stratification by ethnicity and control source indicated no association between the GSTT1 null genotype and hypertension risk. We further confirmed the association by sensitivity analysis. No publication bias was detected.

**Conclusion:**

This meta-analysis suggests that the GSTM1 and GSTT1 null polymorphisms are not associated with the risk of hypertension. Future large well-designed epidemiological studies with individual information, lifestyle factors, and environmental factors are warranted to validate the present findings.

## Introduction

Hypertension, an important worldwide public health challenge, is a major risk factor for cardiovascular disease and end-stage renal damage and it ultimately increases mortality worldwide [1]. Hypertension is generally regarded as a multi-factorial disease that is determined by a combination of genetic factors and environmental stimuli and their interaction [2]. However, the exact pathophysiologic mechanisms underlying the development of hypertension are still unknown.

Oxidative stress, which is due to an imbalance between the generation of reactive oxygen species and the diminished activity of antioxidant enzymes [3], plays an important pathophysiological part in the development of hypertension [4,5]. Free radicals and harmful substances are produced during the process of oxidative stress, which can result in cell membrane lipid peroxidation and damage to DNA and proteins. It has been demonstrated that superoxide anion and hydrogen peroxide production increase, nitric oxide synthesis reduce, and the bioavailability of antioxidants decreases in both experimental and human hypertension [4,5]. Oxidative stress induced by glutathione depletion in normal rats has also been shown to cause and maintain severe hypertension [6]. Glutathione is the most abundant nonprotein intracellular thiol, with multiple roles as an antioxidant agent. GSH is also an important cofactor for different enzymes like glutathione S-transferases (GSTs) [7]. And the GSTs are a family of phase II xenobiotic metabolizing enzymes that protect against endogenous oxidative stress and exogenous potential toxins. The biochemical protection mechanisms by GSTs involve both reduction of organic hydroperoxides, contributing to oxidative stress, and conjugation of electrophilic compounds with glutathione which facilitate their transportation from the cell [8]. The GSTs also can protect cells from oxidative damage, including free radicals produced in the process of the metabolic redox cycle of catechol estrogens [9].

GSTs are found in basically all eukaryotic species and are generally distributed in nature. Eight distinct classes of the soluble cytoplasmic mammalian GST have been identified: a (GSTA), m (GSTM), y (GSTT), p (GSTP), s (GSTS), k (GSTK), o (GSTO), and t (GSTZ) [10]. Two loci in particular, GSTM1 and GSTT1, have received the most attention. The most common variant of the GSTM1 and GSTT1 genes is homozygous deletion (null genotype), which has been associated with the loss of enzyme activity and increased vulnerability to cytogenetic damage [11,12]. Okcu and colleagues [13] found that GSTM1 is one of the genes encoding themu class of enzymes located on chromosome 1p13.3. Daniel [14] reported that the theta class of GST enzymes is encoded by the GSTT1 gene, which is mapped to chromosome 22q11.23. At the GSTM1 locus, one deletion allele and two others (GSTM1a and GSTM1b) have been identified, which differ by C→G substitution [15,16]. The C→G substitution leads to the substitution at amino acid 172 (Lys→ Asn) [16]. And the substitution leads to no functional difference between these two alleles. In result, GSTM1a and GSTM1b are regarded as positive conjugator phenotype. At the GSTT1 locus, two alleles (one functional and the other nonfunctional) have been identified [17]. People with homozygous deletion genotype are grouped into the negative conjugator phenotype, and others are categorized into the positive conjugator phenotype [16]. Previous studies showed that a homozygous deletion, or null genotype, at either the GSTM1 locus or the GSTT1 locus resulted in enzyme function loss, which was hypothesized to be related to risk of hypertension.

A number of case-control studies [18–31] have investigated the relation of null polymorphisms in these two genes to hypertension. However, one thing to be noted is that the results were inconsistent, due to small sample size or other causes. Meta-analysis can be used to pool data from these studies to obtain sufficient statistical power to detect the potential effect of small to moderate sizes associated with these polymorphisms. In this study, we perform a carefully designed and complete meta-analysis to define the effect of GSTM1 and GSTT1 null polymorphisms on the risk for hypertension.

## Methods

### Identification and eligibility of relevant studies

We searched electronic databases, including PubMed and Embase, ISI Web of Science, HuGE Navigator, and Wanfang database of China, for all studies published through August 17, 2013, which had investigated the association between the GSTM1 or GSTT1 genotypes (null genotype vs. wild type) and the risk of hypertension. An updated secondary search was conducted until January 8, 2014, and no relevant new studies were found. The keywords used for searching were glutathione-S-transferase or GST or GSTT1 or GSTM1; polymorphism or genotype; hypertension or essential hypertension or primary hypertension or high blood pressure. References from recent review articles were also checked for relevant articles. We selected all studies that had been published in English. If multiple reports were available for a single unique study population, we included only the most recent or largest reports.

The inclusion criteria were: (1) clear definition of hypertension; (2) studies that examined the association between the GSTM1 or GSTT1 null genotypes and the risk of hypertension; and (3) presentation of original data for the calculation of odds ratios (ORs) with corresponding 95% confidence intervals (95% CIs). The exclusion criteria were: (1) animal studies, case-only studies, case reports, simple commentaries, and review articles; (2) studies with other genotypes of GST; and (3) studies with other diseases. If studies had overlapping subjects, only the study with the largest population was finally selected.

### Data extraction

Characteristics abstracted from the articles included first author, year of publication, country, ethnicity, genotyping method, genotype, definition of hypertension, control source, number of cases, number of controls, gender, age, mean (SD) and adjustment covariates. Two authors (Beihai Ge and Yadong Song) independently extracted the information from every study to minimize the selection bias, and all disagreements about eligibility were resolved during a consensus meeting with a third reviewer.

### Statistical analysis

We investigated the association of GSTM1 and GSTT1 null polymorphisms with hypertension by calculating pooled OR and 95% CI. Two methods were used to estimate between-study heterogeneity across all eligible comparisons: the Cochran’s Q statistic and the I^2^ metric, which quantify between-study heterogeneity irrespective of the number of studies. For the Q statistic, heterogeneity was considered significant if *P* <0.10. A high value of I^2^ indicated a higher probability of the existence of heterogeneity (I^2^ = 0% to 25%, no heterogeneity; I^2^ = 25% to 50%, moderate heterogeneity; I^2^ = 50% to 75%, large heterogeneity; and I^2^ = 75% to 100%, extreme heterogeneity) [32]. Data from the studies were combined using a fixed-effects [33] model or a random-effects [34] model. When heterogeneity was negligible (the corresponding *P* value of Q statistic more than 0.10), a fixed-effects model was performed to obtain the pooled estimator. When between-study heterogeneity was found (the corresponding *P* value of Q statistic below 0.10), a random-effects model was performed. A similar approach was used for performing subgroup analysis by ethnicity. Sensitivity analysis, removing one study at a time, was performed to evaluate the stability of the results. A Galbraith plot was also used to spot the outlier as the possibly major source of between-study heterogeneity [35]. Meta-regression was also performed to study the source of between-study heterogeneity [36]. Publication bias was assessed by Begg’s test [37] and Egger’s test [38] (*P* <0.05 was considered statistically significant). For all analyses, the statistical package Stata 11.0 (Stata Corporation, College Station, Texas, USA) was used. All *P* values were two-tailed with a significant level at 0.05.

## Results

### Characteristics of the studies

**Fig 1:**
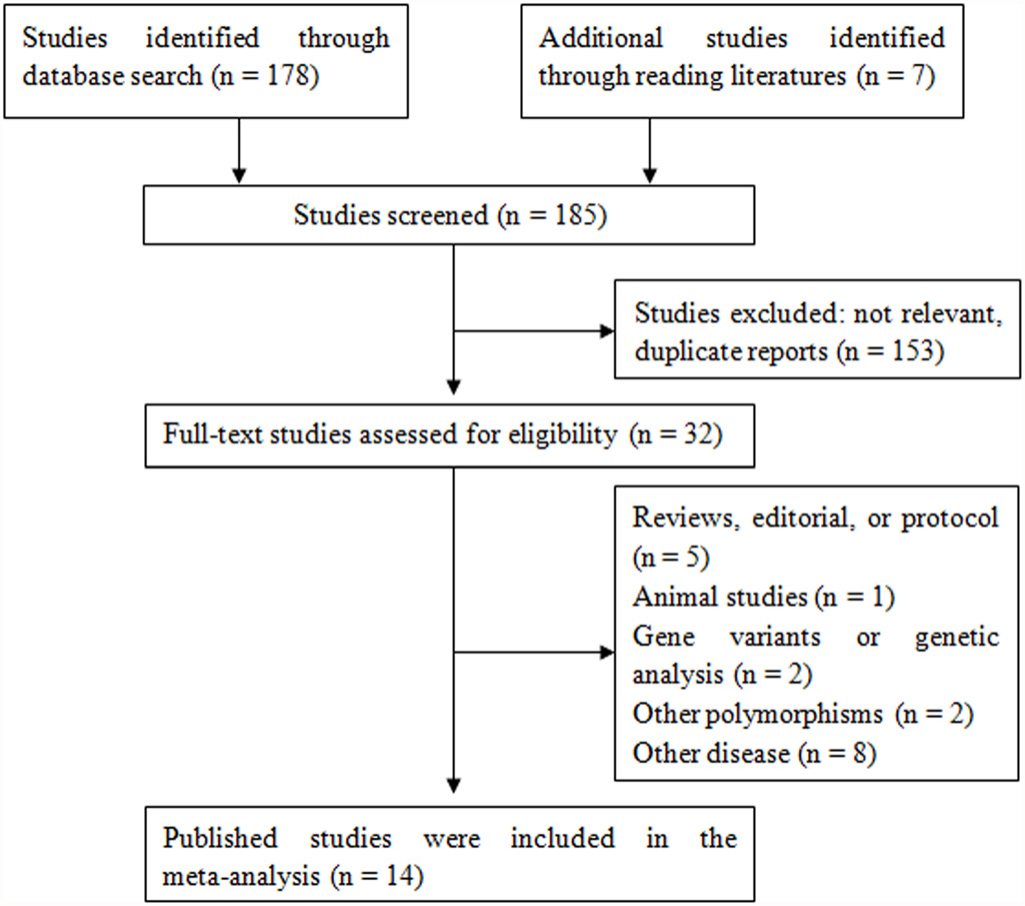
Flow chart depicting exclusion/inclusion of individual studies for meta-analysis.

A total of 185 potentially relevant papers were identified based on the search strategy. The study selection process is shown in Fig. 1. There are 14 studies with 2773 hypertension cases and 3189 controls concerning GSTM1 polymorphism and 13 studies with 2497 hypertension cases and 3078 controls concerning GSTT1 polymorphism. All studies were reported in English. The main study characteristics were summarized in Table 1.

**Table 1:** Characteristics of the studies included in the meta-analysis.

Firstauthor	Year	Country	Ethnicitygroup	Genotyping method	Genotype	Definition	Controlsource	Cases	Controls	Male (%)		Age, year	
										Cases	Controls	Cases	Controls
Bessa [18]	2009	Egypt	African	Multiplex PCR	GSTM1GSTT1	SBP ≥140 mm Hg, DBP ≥90 mmHg, and/or a history of hypertension	PB	40	40	28 (70)	25 (62.5)	54.3 ± 5.6	52.1 ± 6.3
Borah[19]	2011	India	Asian	Multiplex PCR	GSTM1GSTT1	SBP ≥140 mm Hg, DBP ≥90 mmHg, or under antihypertensive medication	PB	223	236	83 (37.2)	94 (39.3)	37.5 ± 12.5	36.6 ± 11.9
Capoluongo[20]	2008	Italy	Caucasian	PCR	GSTM1GSTT1	SBP ≥140 mm Hg, DBP ≥90 mmHg, or were receiving any of the antihypertensive medications	PB	255	99	82 (32.1)	36 (36.3)	85.4 ± 4.6	87.0 ± 5.3
Hussain [22]	2012	United ArabEmirates	Asian	Multiplex PCR	GSTM1GSTT1	SBP ≥140 mm Hg, DBP ≥90 mmHg, or current use of anti-hypertensive medication	HB	30	33	NA	NA	40.1 ± 14	41.7 ± 13.6
Marinho [25]	2006	Portugal	Caucasian	Multiplex PCR	GSTM1GSTT1	NA	PB	94	207	57 (60.6)	NA	67.4 ± 10	52.6 ± 12
Oniki [27]	2008	Japan	Asian	PCR	GSTM1GSTT1	SBP ≥140 mm Hg, DBP ≥90 mmHg, and/or a history of hypertension	PB	130	338	100 (76.9)	226 (66.8)	56.4 ± 8.4	52.0 ± 9.1
Polimanti [28]	2011	Italy	Caucasian	Multiplex PCR	GSTM1GSTT1	Based on the physician’s diagnosis and on the use of antihypertensive medications	PB	193	210	80 (41.5)	74 (35.2)	58.7 ± 0.7	57.6 ± 0.8
Turkanoglu [29]	2010	Turkey	Caucasian	Multiplex PCR	GSTM1GSTT1	SBP ≥140 mm Hg, DBP ≥90 mmHg, and/or use of antihypertensive drugs	HB	150	127	NA	NA	35–90	35–90
Lee [24]	2012	Korea	Asian	Multiplex PCR	GSTM1GSTT1	SBP ≥140 mm Hg, DBP ≥90 mmHg	PB	258	497	NA	NA	NA	NA
Wang [30]	2012	China	Asian	PCR	GSTM1GSTT1	NA	PB	323	202	NA	NA	NA	NA
Yang [31]	2004	Taiwan	Asian	Multiplex PCR	GSTM1GSTT1	NA	HB	475	230	241 (50.7)	110 (47.8)	NA	NA
Miranda-Vilela [26]	2010	Brazil	Mixed	PCR	GSTM1GSTT1	SBP ≥140 mm Hg, DBP ≥90 mmHg	PB	91	110	46 (50.5)	54 (49.1)	50.5±1.2	50.6±01.2
Jiang [23]	2010	USA	Mixed	PCR	GSTM1GSTT1	History of hypertension, medication use	HB	278	812	NA	NA	25–64	25–64
Cruz-Gonzalez [21]	2009	Spain	Caucasian	PCR	GSTM1	SBP ≥140 mm Hg, DBP ≥90 mmHg	PB	281	110	120 (42.7)	51 (46.4)	64.1	63.4±15.7

Abbreviations: BMI: body mass index; DBP: diastolic blood pressure; GSTM1: glutathione S-transferase M1; NA: not available; GSTT1: glutathione S-transferase T1; HB: hospital based; PB: population based; PCR: polymerase chain reaction; SBP: systolic blood pressure; SD: standard deviation.

### Pooled analyses

**Fig 2:**
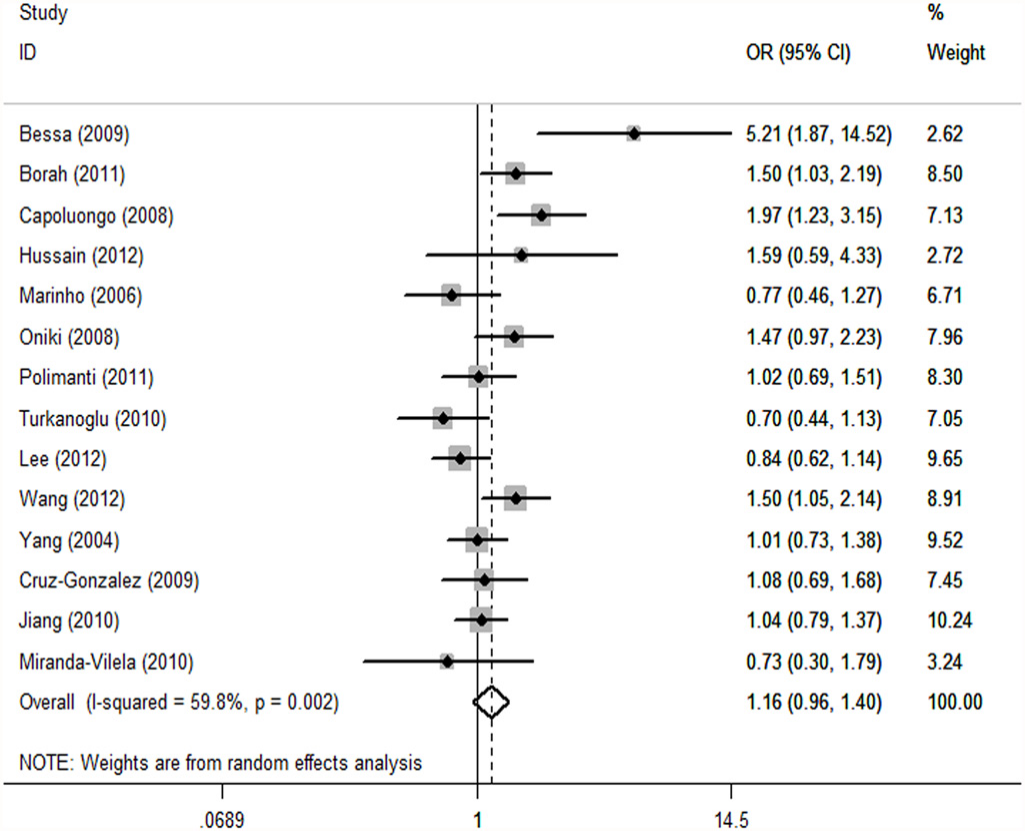
Forest plot of the GSTM1 null genotype and risk of hypertension in overall analysis. The squares indicate the odds ratios in the individual studies; each square’s size is proportional to the weight of the corresponding study in the meta-analysis. The diamond indicates the pooled odds ratio. Horizontal lines represent 95% confidence interval. The unbroken vertical line is at the null value (OR = 1.0).

**Fig 3:**
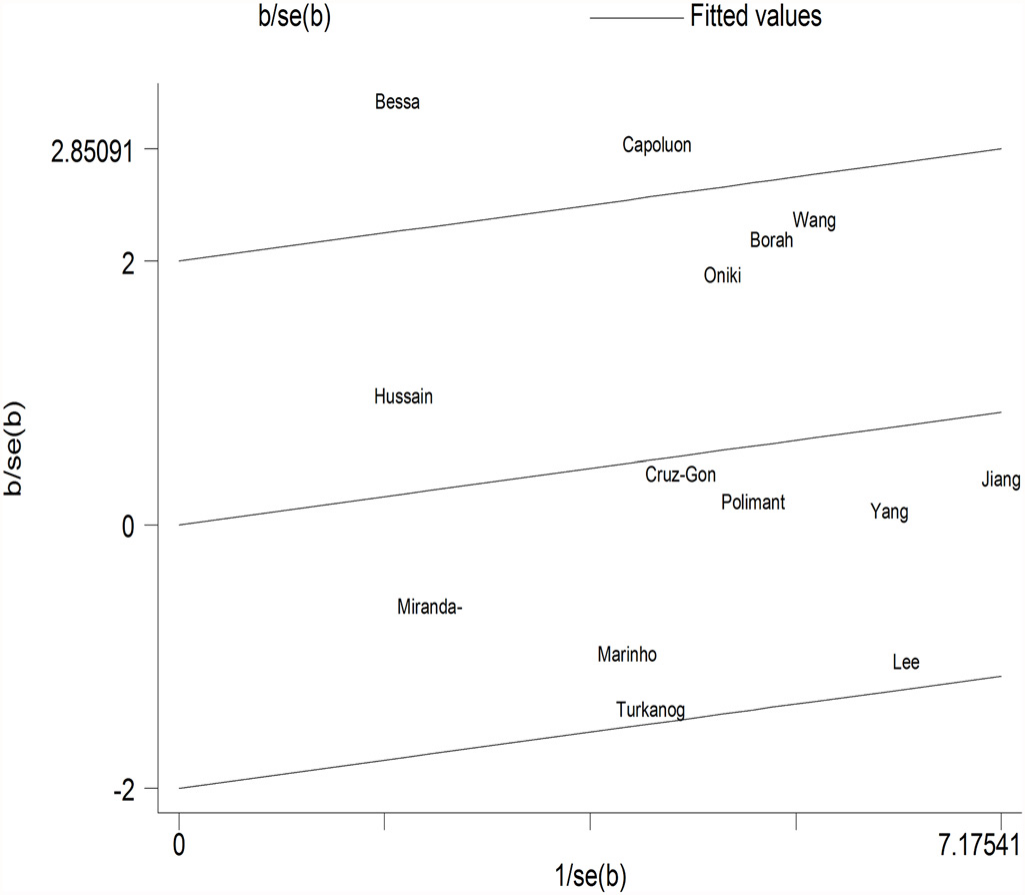
Galbraith plot of meta-analysis of the association between GSTM1 null genotype and hypertension.

GSTM1. Because substantial between-study heterogeneity was observed (I^2^ = 59.8%), we used a random-effects model. The pooled estimator for GSTM1 null suggested that it might show a similarly small increased risk for hypertension, although the effect was not statistically significant (OR = 1.16, 95% CI: 0.96, 1.40; I^2^ = 59.8%, *P* = 0.002) (Fig. 2). Two studies were spotted by Galbraith plot as possible major sources of heterogeneity (Fig. 3). After adjustment for heterogeneity by omitting these 2 studies [18,20], moderate heterogeneity was found (I^2^ = 37.2%) and meta-analysis also showed no significant association between this polymorphism and hypertension risk (OR = 1.07, 95% CI: 0.92, 1.24) (Table 2). When stratifying for ethnicity, an OR of 1.22 (95% CI: 0.97, 1.54; I^2^ = 51.9%) and 1.04 (95% CI: 0.74, 1.45; I^2^ = 64.3%) resulted for the null genotype, among Asians and Caucasians, respectively (Table 2). When control source subgroups were considered, the OR was 0.98 (95% CI: 0.82, 1.18) in hospital based persons compared to 1.25 (95% CI: 0.98, 1.60) in population based persons (Table 2).

**Table 2:** Summary of meta-analysis of studies examining GSTM1 and GSTT1 polymorphisms and hypertension risk.

Studies	Number of studies	Test of association		Test of heterogeneity		Effect model	Publication bias
		OR	95% CI	*P* value	I^2^ (%)		*P* value (Egger’s)
GSTM1 polymorphism							
All studies	14	1.16	0.96, 1.40	0.002	59.8	Random	0.29
All studies(adjustment for heterogeneity ^a^)	12	1.07	0.92, 1.24	0.093	37.2	Random	0.90
Ethnicity							
Asian	6	1.22	0.97, 1.54	0.065	51.9	Random	0.35
Caucasian	5	1.04	0.74, 1.45	0.025	64.3	Random	0.83
Control							
HB	4	0.98	0.82, 1.18	0.39	0.8	Fixed	0.91
PB	10	1.25	0.98, 1.60	0.002	65.6	Random	0.37
GSTT1 polymorphism							
All studies	13	1.14	0.88, 1.53	0.000	80.3	Random	0.34
All studies(adjustment for heterogeneity ^b^)	7	1.18	0.98, 1.43	0.723	0.0	Fixed	0.40
Ethnicity							
Asian	6	1.09	0.72, 1.65	0.000	84.1	Random	0.32
Caucasian	5	1.05	0.57, 1.94	0.000	82.6	Random	0.41
Control							
HB	4	0.92	0.6, 1.4	0.032	65.8	Random	0.48
PB	9	1.23	0.84, 1.8	0.000	82.8	Random	0.61
Both null polymorphisms	6	1.23	0.49, 3.10	0.000	84.9	Random	0.08

Abbreviations: GSTT1: glutathione S-transferase T1; GSTM1: glutathione S-transferase M1; OR: odds ratio; CI:Confidence interval.

^a^ Adjustment for heterogeneity was performed by excluding 2 studies as the outliers spotted by Galbraith plot and the possible major source of heterogeneity.

^b^ Adjustment for heterogeneity was performed by excluding 6 studies as the outliers spotted by Galbraith plot and the possible major source of heterogeneity.

**Fig 4:**
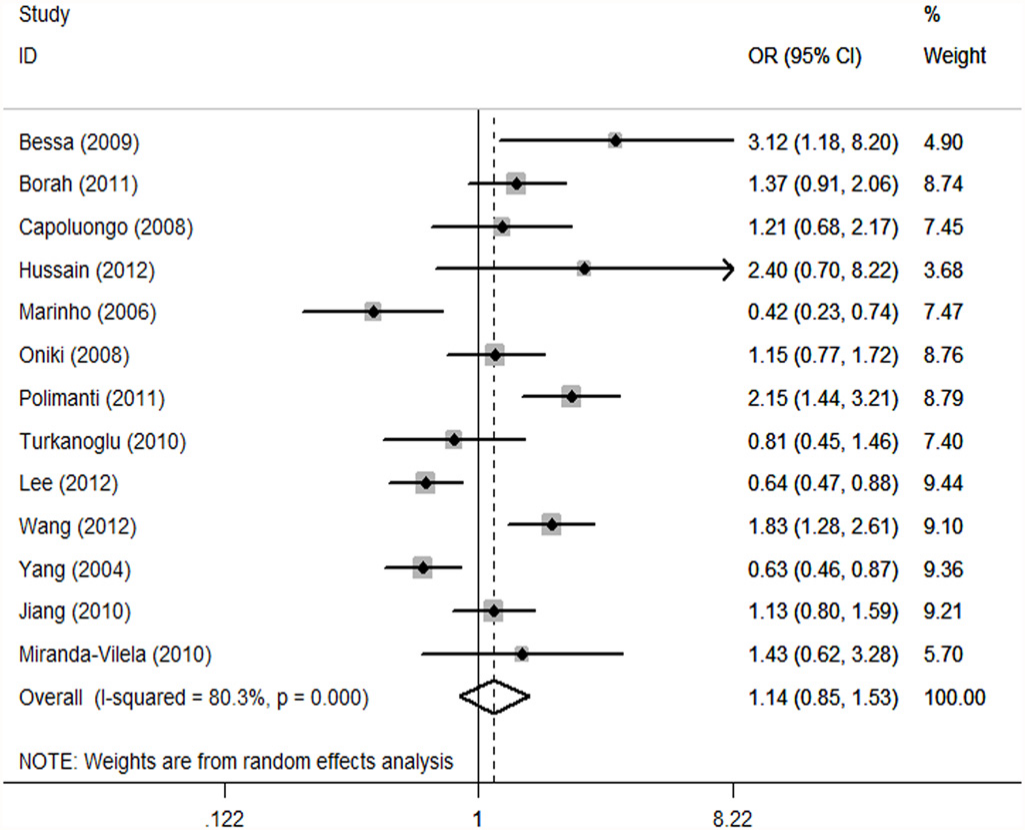
Forest plot of the GSTT1 null genotype and hypertension risk in overall analysis. The squares indicate the odds ratios in the individual studies; each square’s size is proportional to the weight of the corresponding study in the meta-analysis. The diamond indicates the pooled odds ratio. Horizontal lines represent 95% confidence interval. The unbroken vertical line is at the null value (OR51.0).

**Fig 5:**
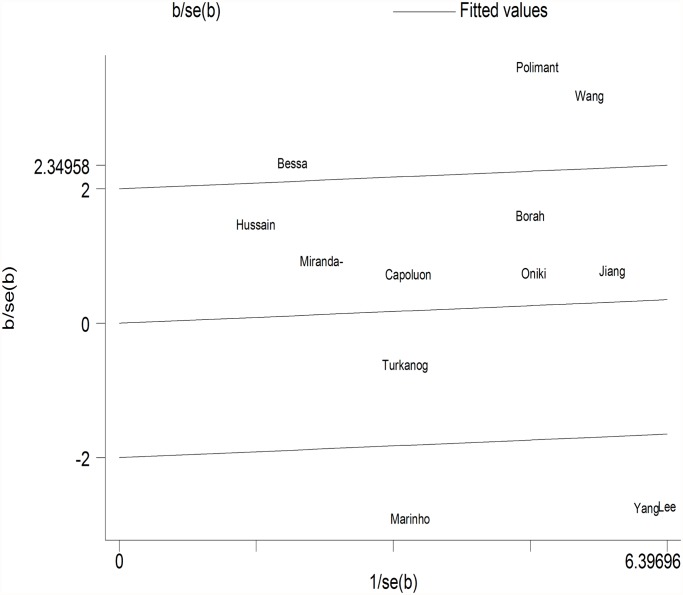
Galbraith plot of meta-analysis of the association between GSTT1 null genotype and hypertension.

GSTT1. As substantial between-study heterogeneity was observed with GSTT1 (I^2^ = 80.3%), we again used a random-effects model. The meta-analysis resulted in a statistically non-significant association between GSTT1 null genotype and hypertension. The overall OR was 1.14 (95% CI: 0.85, 1.53; I^2^ = 80.3%, *P* = 0.000) (Fig. 4). After adjustment for heterogeneity by omitting these six studies [18,24,25,28,30,31] (Fig. 5), no heterogeneity was found (I^2^ = 0%) and meta-analysis also showed no significant association between this polymorphism and hypertension risk (Table 2). No significant association was found in stratified analyses according to ethnicity or source of controls. The OR was 1.05 (95% CI: 0.57, 1.94) in Caucasians and 1.09 (95% CI: 0.72, 1.65) in Asians (Table 2). When stratifying for source of controls, an OR of 0.92 (95% CI: 0.60, 1.40) and 1.23 (95% CI: 0.84, 1.80) resulted for the null genotype, among hospital based and population based persons, respectively (Table 2).

For gene-gene interaction, we assessed the association between both the null genotype of GSTs and risk of hypertension. The data on both the null genotypes of GSTs among cases and controls were available in five studies, which included 1427 cases and 896 controls. The meta-analysis resulted in a statistically non-significant association between both the null genotypes of GSTM1 and GSTT1 and hypertension (OR = 1.23, 95% CI: 0.49, 3.10) (Table 2).

### Sensitivity analyses and publication bias

We sequentially deleted data from single studies involved in the meta-analysis to investigate the impact of individual data sets on the pooled OR. For GSTM1, the I-square value ranged from 49.3% to 62.9% when any single study was omitted, with the result that the overall effect size was unchanged. A sensitivity analysis yielded a range of ORs from 1.11 (95% CI: 0.93, 1.33) to 1.20 (95% CI: 0.99, 1.45). For GSTT1, the I-square value varied between 77% and 81.9% when any single study was removed, with the result that the overall effect size was not altered. No individual study had an undue influence on the pooled ORs, and the between-study heterogeneity still existed when any single study was excluded. A sensitivity analysis yielded little change in the observed risk estimates, which shifted from 1.06 (95% CI: 0.80, 1.42) to 1.22 (95% CI: 0.92, 1.64). Hence, we can conclude that the results of the studies concerning both GSTM1 and GSTT1 are stable and credible.

**Fig 6:**
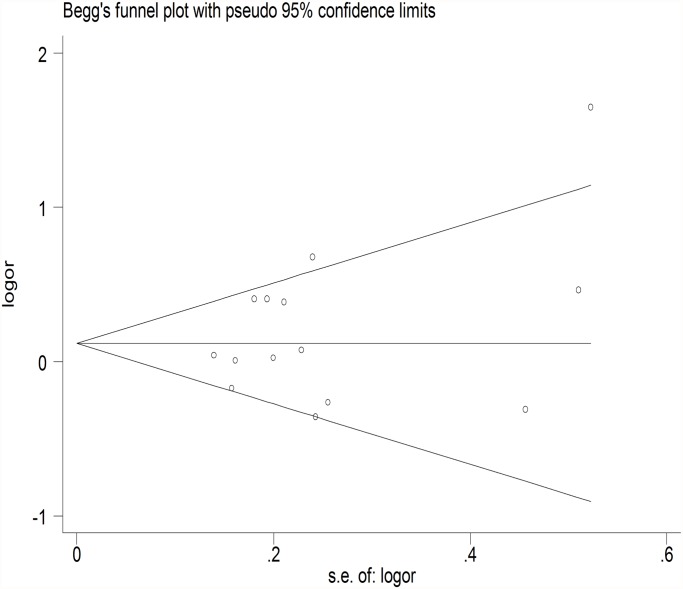
Begg’s funnel plot of the association between GSTM1 null genotype and hypertension. Each dot represents a separate study for the indicated association. Location outside the delineated triangle (pseudo 95% confidence intervals) suggests a publication bias.

**Fig 7:**
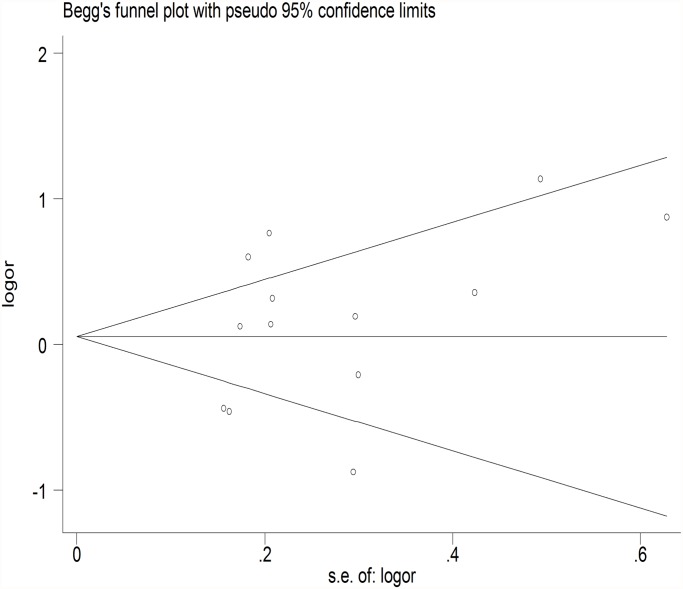
Begg’s funnel plot of the association between GSTT1 null genotype and hypertension. Each dot represents a separate study for the indicated association. Location outside the delineated triangle (pseudo 95% confidence intervals) suggests a publication bias.

The meta-regression was conducted with the introduction of covariates including publication year, ethnicity, genotyping method, sample size, controls source and essential hypertension. However, no covariate was identified as a potential source of between-study heterogeneity for any comparison. A Begg’s funnel plot and Egger’s test were performed to assess the publication bias. The shape of the funnel plot did not reveal any evidence of obvious asymmetry (Fig. 6) (Fig. 7), and the Egger’s test was then used to provide statistical evidence of funnel plot symmetry. The results still did not demonstrate any evidence of publication bias in any studies (Table 2).

## Discussion

The present meta-analysis examined the effect of GSTM1 and GSTT1 null polymorphisms on the risk of hypertension. To our knowledge, a previous meta-analysis [39] has discussed on the association between GSTM1 and GSTT1 polymorphisms and risk of hypertension. However, the evidence from this article was limited. First, when between-study heterogeneity was found, random-effects model should be performed. So, pooled estimate of odds ratios in previous meta-analysis was inappropriate because they used fixed-effects model. Second, two studies [24,31] should also be included in the meta-analysis. Third, the previous meta-analysis did not perform stratified analyses and meta-regression to identify possible sources for the observed heterogeneity. These weaknesses decreased the power of results. Trying to generate more exact conclusions, we conducted this meta-analysis that included 14 case—control studies. Our results indicated no statistically significant association between GSTM1 or GSTT1 null polymorphism (null versus non-deleted) and risk of hypertension. When studies were stratified by ethnicity and control source, we still did not find significant associations. There were also a number of potentially interesting gene-gene and gene-environment interactions reported in individual studies. However, these were too few and too inconsistent to allow a meta-analysis.

Our results are different from previous meta-analysis [39]. The previous meta-analysis revealed a significant association between the null genotype of GSTT1 (OR = 1.30; 95% CI: 1.13, 1.50; *P* = 0.000) and risk of hypertension. However, when including two additional studies [24,31] which have strong reverse associations for GSTT1, the present meta-analysis revealed statistically non-significant association between GSTT1 null genotype and hypertension risk (OR = 1.14, 95% CI: 0.85, 1.53; *P* = 0.000). Concerning GSTM1, the previous meta-analysis implied that GSTM1 null genotype was positively associated with the risk of hypertension (OR = 1.22; 95% CI: 1.08, 1.39; *P* = 0.002). However, our results suggested that the effect was not statistically significant (OR = 1.16, 95% CI: 0.96, 1.40; *P* = 0.002).

Assessment of heterogeneity is necessary for most meta-analyses [40,41]. Heterogeneity could result from genotyping error, selection bias, population stratification, gene-gene or gene-environment interaction, allelic heterogeneity, or chance [41,42]. The I^2^ values surpassed the threshold of 50% indicated the presence of heterogeneity and insufficient power [43]. The present meta-analysis showed significant heterogeneity in the results for GSTM1 and GSTT1. Concerning GSTM1, two studies [18,20] was spotted by Galbraith plot and may be the possible major source of heterogeneity. The average age of people in one study [20] was more than 80. The other study [18] was the only study that conducted in Africa. Santovito et al. [44] demonstrated that changes in GSTM1-null prevalence had been documented for ethnicity and aging. This may be the reason why the two studies determine most of heterogeneity. The heterogeneity may be caused by methodological differences across studies. The potential selection bias, which may have been introduced by a poorly defined study base, was a usual methodological concern of the studies reviewed [45]. If case-control studies fail to properly sample from the base, the controls do not reflect the exposure and/or genotype distributions of the source population, and the results are biased. However, they might also indicate ethnic differences in the contribution of these genotypes to hypertension risk, and if so, summary ORs for these genotypes could be misleading. What is more, because of limited knowledge on how much heterogeneity resulted from errors and biases, the summary estimates provided in this meta-analysis would reflect only a crude analysis. Further studies need to focus on exploring the sources of heterogeneity.

GST genetic polymorphisms imply variations in enzyme activities that can result in oxidative stress susceptibility through alterations in GSH metabolism [46]. The homozygous deletion of these loci has been reported to be associated with loss of enzyme function. The null genotype of GSTM1 has been suggested to be associated with the risk of a number of diseases, including alcoholic liver disease [47] and asthma [48]. However, previous meta-analyses showed that the GSTM1 polymorphism was not associated with the risk of rheumatoid arthritis [49]. The null genotype of GSTT1 has been implicated in the genesis of several diseases, including asthma [48] and type 2 diabetes [50], but not coronary heart disease [51] or COPD [52]. Recently, Delles et al. did not find an association between GSTM gene variants and hypertension [53]. Present meta-analysis showed that there was no significant association between GSTT1 and GSTM1 null genotypes and hypertension risk. It seems that GSTT1 and GSTM1 polymorphisms cannot influence the risk of hypertension in all studies.

In this study, although we pooled all published studies currently available on this topic, we believe our study is still far from conclusive. The association of genetic polymorphisms with hypertension is strongly influenced by differences in the selection of cases and controls, ethnicity, sample size, environmental factors and other ecological factors. Selection bias is also possible in hospital-based studies because the GSTs may be related to the risk for chronic diseases. And most studies were not clear about whether cases represented the first diagnosis of hypertension. Further studies with unbiased-matched homogeneous patients and well matched controls are required to examine associations between the GSTM1 and GSTT1 polymorphisms and hypertension risk. Furthermore, the negative result also might be caused by interethnic differences. Changes in GSTM1-null or GSTT1-null prevalence have been documented for ethnicity and aging [44]. According to a report [54], the frequency of the GSTM1 null genotype is 53% in Caucasians, 27% in African-American subjects, and 53% in Asians. The frequency of the GSTT1gene deletion is 20% and 47% for Caucasians and Asians, respectively. Finally, GST variants often exert their effects through interaction with environmental exposures (e.g., cigarette smoking, alcohol consumption). However, most studies did not provide the stratified data. Larger studies will be needed to explore potential interactions between GST polymorphisms and environmental oxidative exposures. These design issues would have contributed to heterogeneous results in the literature. Future large well-designed epidemiological studies with individual information, lifestyle factors, and environmental factors are warranted to validate the present findings.

Several limitations existed in our meta-analysis. First, the present analyses were based on unadjusted estimates because most studies did not provide adjusted data. More precise analysis including individual data, lifestyle factors, and environmental factors, should be conducted if possible. Second, the present analyses were based upon a thousand cases and a thousand controls. In several studies, the sample sizes in our meta-analyses were small, which did not have enough power to provide a confirmed conclusion. Third, because most of the included studies were conducted on Asians and a few on Caucasians, we must interpret the results carefully. Further studies concerning populations in other areas such as Africa and North America are required to diminish the ethnic variation-produced biases. Finally, although the present meta-analysis of all studies and a set of subgroup analyses have been undertaken, significant heterogeneity still persisted, limiting the interpretation of pooled risk estimates.

Despite the limitations, this meta-analysis suggests that the GSTM1 and GSTT1 null polymorphisms are not associated with the risk of hypertension. Future large well-designed epidemiological studies are warranted to validate the present findings.

## Supporting Information

S1 PRISMA Checklist PRISMA 2009 checklist in this meta-analysis. (DOC)
